# First-principles study of crystallographic slip modes in *ω*-Zr

**DOI:** 10.1038/s41598-017-09153-w

**Published:** 2017-08-21

**Authors:** Anil Kumar, M. Arul Kumar, Irene J. Beyerlein

**Affiliations:** 1 Theoretical Division, Los Alamos National Laboratory, Los Alamos, NM 87545 USA; 2 Materials Science and Technology Division, Los Alamos National Laboratory, Los Alamos, NM 87545 USA; 3 Mechanical Engineering Department, Materials Department, University of California at Santa Barbara, Santa Barbara, CA 93106 USA

## Abstract

We use first-principles density functional theory to study the preferred modes of slip in the high-pressure *ω* phase of Zr. The generalized stacking fault energy surfaces associated with shearing on nine distinct crystallographic slip modes in the hexagonal *ω*-Zr crystal are calculated, from which characteristics such as ideal shear stress, the dislocation Burgers vector, and possible accompanying atomic shuffles, are extracted. Comparison of energy barriers and ideal shear stresses suggests that the favorable modes are prismatic 〈*c*〉, prismatic-II $\langle 10\bar{1}0\rangle$ and pyramidal-II 〈*c* + *a*〉, which are distinct from the ground state hexagonal close packed *α* phase of Zr. Operation of these three modes can accommodate any deformation state. The relative preferences among the identified slip modes are examined using a mean-field crystal plasticity model and comparing the calculated deformation texture with the measurement. Knowledge of the basic crystallographic modes of slip is critical to understanding and analyzing the plastic deformation behavior of *ω*-Zr or mixed *α*-*ω* phase-Zr.

## Introduction

Due to﻿ an outstanding ﻿resistance to corrosion and radiation damage, Zr and its alloys have become an attractive class of metals for use in aggressive environments, such as those found in chemical and nuclear reactors^1–3^. Consequently, for several decades the mechanical behavior of Zr as a function of temperature and pressure has been the subject of intense research. At ambient pressure, the stable phase of Zr has a hexagonal close-packed (hcp) structure (*α*-phase). Moderately high pressures, in the range of 2–4 GPa, however, can cause a structural phase transformation to a simple hexagonal (hex) structure (the *ω*-phase)^2, 4^. This transformation can occur under pressures generated either by static deformation^5–7^, shock loading^8–11^, or high-pressure torsion (HPT)^12^. The *α* phase is often not completely recovered when returning to ambient temperature and pressure from the loaded, pressurized state. The amount of *ω* phase retained can be substantial and depend on the loading conditions. For example, shock loading retains nearly 80% *ω* phase after unloading to ambient pressure^13^ whereas HPT, which involves shearing under high pressures, retains nearly 100% *ω* phase^12^. To date, understanding the strength of the *ω*-Zr phase or mixed *α*-*ω* phase Zr is severely limited. Fundamental knowledge of the preferred crystallographic slip systems in *ω*-Zr is currently lacking and prevents further insight needed for modeling and designing of Zr and Zr alloys in service conditions^14–17^.

Over the years, basic knowledge of the preferred crystallographic slip modes in hcp *α*-Zr has been developed through direct observation of dislocations in motion, post-mortem dislocation analyses, and investigations of texture evolution and stress-strain response during mechanical deformation^18–20^. These measurements have been complemented by numerical studies over similar size and time scales, such as ab-initio and atomic-scale calculations of energies involved in the creation and motion of dislocations on particular slip planes, dislocation mechanics simulations of paired dislocation interactions and single crystal hardening, and micromechanics calculations (e.g., effective-medium polycrystal schemes) of microstructural evolution and stress-strain response^21–28^. Obtaining similar data on *ω*-Zr, however, is challenging due to the need to directly observe and measure dislocation glide under pressure^29^. The favored modes of crystallographic slip strongly depend on the particular electronic and atomic structure of the material and, hence, those for hex *ω*-Zr should be distinct from hcp *α*-Zr.

As a way of identifying the preferred slip modes, here we calculate using first-principles density functional theory (DFT) the generalized stacking fault energy (GSFE) surfaces^30, 31^ for several possible, geometrically admissible slip modes in *ω*-Zr. The GSFE surface is the excess energy per unit area for a given relative displacement vector *u* of one half of the crystal with respect to the other half when a perfect crystal is cut across the slip plane into two parts^32^. In this way, it provides a calculation of the variation in energy corresponding to the shearing action caused by the glide of a dislocation on specific crystallographic planes. From these calculations, estimates can be attained for the ideal shear stress that would resist the shear on a particular mode of slip, the energetically favorable shear displacement paths, and any extra local atomic shuffling and glide, outside of the theoretical glide direction. The more likely modes on which dislocations would glide in an actual metal would tend to possess the lower energy barriers for shear or ideal shear stresses. This analysis, however, is a relative one, in the sense that the actual stress barriers that would resist the motion of a dislocation along these pathways at deformation temperatures and pressures are typically three to four orders of magnitude lower than the ideal shear stress at 0 K. Based on the GSFE calculations, we show that the favored crystallographic slip modes in *ω*-Zr are prismatic 〈*c*〉, prismatic-II $\langle 10\bar{1}0\rangle$ and pyramidal-II 〈*c* + *a*〉. Together these three modes can accommodate any arbitrary deformation state applied to the crystal and notably are distinct from the two preferred modes in *α*-Zr, being prismatic 〈*a*〉 and pyramidal-I 〈*c* + *a*〉^33^. Since *ω*-Zr is a high-pressure phase, we also study the effect of pressure on the energetics of these slip modes and find that the ones favored at ambient pressures are the same as those favored at high pressure. In order to examine the possibility of activating the identified slip modes in *ω*-Zr, we employ the mean-field visco-plastic self-consistent (VPSC) model to simulate the deformation texture under compression. In the VPSC calculation, the ratios between the CRSS values among the different slip modes are adjusted to match the predicted texture with the measurements from Wenk *et al*.^2^ The VPSC results confirm the possibility of activating the identified slip modes, and also suggest that they do not possess similar resistances to activation.

## Results

DFT calculations are first carried out to calculate the lattice and elastic constants of *ω* and *α* phases of Zr. Table 1 lists the results of these calculations as well as those from another DFT calculation^34^ and available from measurement^1, 6, 35^. Comparison finds that the calculated values of the lattice constants *a* and *c* for both the *α* and *ω* phases are in very good agreement with experimentally measured values. Calculated elastic constants for the *α* phase are a﻿lso in good agreement with the experimental values. Experimental values of the elastic constants for the *ω* phase are, however, not available and hence the calculations reported here await validation.

**Table 1:** Calculated lattice and elastic constants for the *ω* and *α* phases of Zr at P = 0 and 4.9 GPa.

Phase	P	a (*Å*)	c (*Å*)	C_11_	C_33_	C_12_	C_13_	C_44_	C_66_	*R*_1_, *R*_2_, *R*_3_	References
*ω*	0	5.032	3.152	160.7	197.2	78.8	52.0	34.8	40.9	0.81, 1.18, 2.03	This Work
	0	5.036	3.109								Ref. 6 [Expt.]
	0	5.036	3.152	161.7	195.6	72.6	53.5	33.7			Ref. 34 [Calc.]
	4.9	4.954	3.106	174.9	216.5	89.9	59.3	35.7	42.5	0.81, 1.19, 2.14	This Work
*α*	0	3.231	5.174	135.1	166.1	80.3	70.7	26.1	27.4	0.69, 1.05, 1.68	This Work
	0	3.233^1^	5.146^1^	144.0^35^	166.0^35^	74.0^35^	67.0^35^	33.0^35^	35.0^35^	0.78, 1.06, 1.42	Expt.
	4.9	3.177	5.105	139.6	183.3	94.5	80.6	24.1	22.6	0.63, 0.93, 1.91	This Work

Elastic constant values are in GPa. The elastic anisotropy ratio for the HCP metals is given by ref. 61: ${R}_{1}=\frac{{C}_{11}+{C}_{12}-{C}_{33}}{{C}_{13}}$, ${R}_{2}=\frac{{C}_{66}}{{C}_{44}}$, ${R}_{3}=\frac{{C}_{33}+{C}_{11}+{C}_{12}-{C}_{13}\times \sqrt{{R}_{1}^{2}+8}}{4{C}_{44}}$.

These calculations were repeated at a higher pressure of 4.9 GPa. This pressure was selected since it lies above the phase transition pressure to *ω*-Zr and corresponds to one at which polycrystalline Zr is observed to be fully transformed to the *ω*-phase^2^. The calculations here find that under pressure the lattice constants decrease and the elastic constants increase for both the *α* and *ω* phases of Zr. Using the values in Table 1, the elastic anisotropy measure *R*_1_, *R*_2_, *R*_3_ defined in Table 1 for *ω*-Zr are 0.81, 1.18, 2.03, which is higher than those for *α*-Zr, which are 0.69, 1.05, 1.68 at zero pressure. All measures suggest that intrinsically *ω*-Zr is more elastically anisotropic than *α*-Zr.

**Figure 1:**
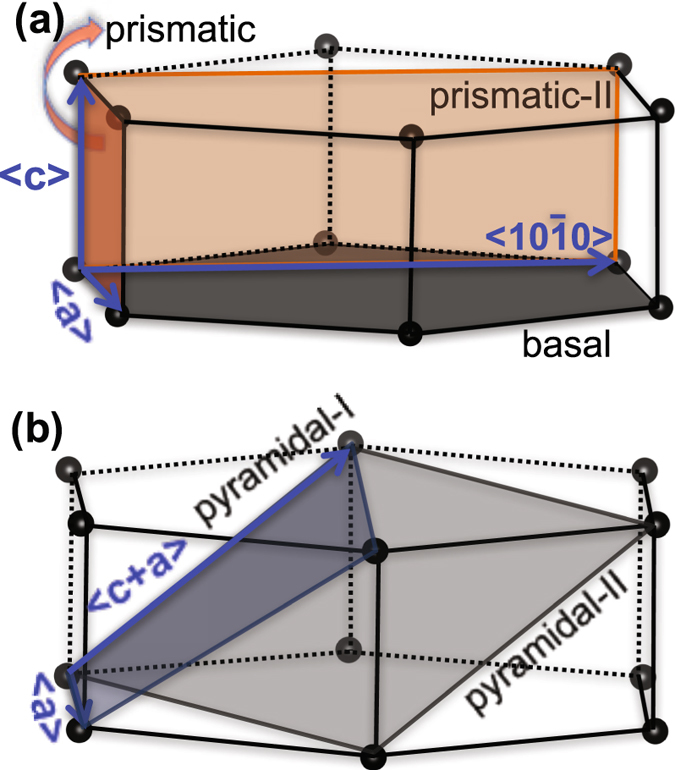
Schematic showing the possible slip modes on different planes in *ω*-Zr. (**a**) basal 〈*a*〉 and basal $\langle 10\bar{1}0\rangle$ slip modes on the basal plane; prismatic 〈*a*〉 and prismatic 〈*c*〉) slip modes on the prismatic plane; and prismatic-II $\langle 10\bar{1}0\rangle$ and prismatic-II 〈*c*〉 slip modes on the prismatic-II plane, (**b**) pyramidal-I 〈*a*〉 and pyramidal-I 〈*c* + *a*〉 slip modes on the pyramidal-I plane; and pyramidal-II 〈*c* + *a*〉 slip modes on the pyramidal-II plane.

For the low-symmetry hex crystal structure, we define several possible slip modes, accommodating both deformation in the 〈*a*〉 and 〈*c*〉 directions. Their orientation and directions with respect to the hex unit cell are shown in Fig. 1. Altogether, these nine modes span five distinct planes and four of these planes are associated with two independent slip modes. Each mode is comprised of multiple slip systems that are crystallographically identical but independently oriented. The number of slip systems per mode are determined by considering all hex crystal symmetries. Table 2 gives the crystallography of these modes and the number of independent slip systems belonging to each. The group of slip modes accommodating 〈*a*〉 deformation are denoted as basal 〈*a*〉, basal $\langle 10\bar{1}0\rangle$, prismatic 〈*a*〉, prismatic-II $\langle 10\bar{1}0\rangle$, and pyramidal-I 〈*a*〉, and the group accommodating 〈*c*〉 deformation are referred to as prismatic 〈*c*〉, prismatic-II 〈*c*〉, pyramidal-I 〈*c* + *a*〉, and pyramidal-II 〈*c* + *a*〉.

**Table 2:** Details of the supercells used in the calculation of the GSFE curves for each slip mode.

Index	Plane	Slip Mode	*L*_*x*_ × *L*_*y*_ × *L*_*z*_	No. of Atoms	No. of Layers	Indep. Slip System	Compact/Extended
1	basal	{0001} $\langle 1\bar{2}10\rangle$	5.032 × 8.716 × 46.52	66	22	3	Compact
		{0001} $\langle 10\bar{1}0\rangle$	$(\mathrm{[1}\bar{2}\mathrm{10]}\times \mathrm{[10}\bar{1}\mathrm{0]}\times \mathrm{[0001]})$			3	Extended
2	prismatic	$\mathrm{\{10}\bar{1}\mathrm{0\}}$ 〈0001〉	5.032 × 3.152 × 67.29	42	42	3	Compact
		$\mathrm{\{10}\bar{1}\mathrm{0\}}\,\langle 1\bar{2}10\rangle$	$(\mathrm{[1}\bar{2}\mathrm{10]}\times \mathrm{[0001]}\times \mathrm{[10}\bar{1}\mathrm{0]})$			3	Compact
3	prismatic-II	$\{\bar{1}2\bar{1}\mathrm{0\}}$ 〈0001〉	8.716 × 3.152 × 65.32	66	22	3	Compact
		$\{\bar{1}2\bar{1}\mathrm{0\}}\,\langle 10\bar{1}0\rangle$	$(\mathrm{[10}\bar{1}\mathrm{0]}\times \mathrm{[0001]}\times \mathrm{[1}\bar{2}10])$			6	Extended
4	pyramidal-I	$\mathrm{\{10}\bar{1}\mathrm{1\}}\,\langle 1\bar{2}10\rangle$	5.032 × 10.757 × 54.40	84	14	6	Extended
		$\mathrm{\{10}\bar{1}\mathrm{1\}}\,\langle 11\bar{2}3\rangle$	$(\mathrm{[1}\bar{2}\mathrm{10]}\times \mathrm{[10}\bar{1}\mathrm{2]}\times [\bar{1}\mathrm{011]})$			12	Extended
5	pyramidal-II	$\mathrm{\{11}\bar{2}\mathrm{2\}}\,\langle 11\bar{2}3\rangle$	8.716 × 5.938 × 46.52	66	22	6	Extended
			$$(\mathrm{[10}\bar{1}\mathrm{0]}\times \mathrm{[11}\bar{2}\mathrm{3]}\times \mathrm{[11}\bar{2}\mathrm{2]})$$				

The fourth column shows the dimensions (in *Å*) and the crystallography of the supercell along the x, y and z directions. The sixth column shows the number of layers in the model normal to the slip plane and the eighth column shows whether the full dislocation is compact or extended in a dissociated state.

Supercells that are periodic in the x, y and z directions are adopted for the DFT calculations. The periodic dimension along the z direction contains a 15 *Å*-thick vacuum layer. The values of *a* and *c* in *ω*-Zr are used to construct the supercell. Since the dimensions of the unit cell depend on the slip plane, the appropriate number of atoms and the dimensions of the supercells had to be determined for each plane (see Fig. S1). Table 2 lists the number of atoms, the dimensions along x, y, and z of the supercells for each plane and the number of atomic layers the supercell encases. For each plane, the supercell dimensions corresponded to the minimum number of layers for which convergence in system energy is attained.

Particularly in low symmetry crystal structures like hcp and hex, glide planes among the various slip modes are topologically different, some can be rumpled and the atomic positions about the glide direction can be asymmetric^36^. In such cases, additional mechanisms, like local atomic shuffling and/or glide non-parallel to the theoretical glide direction, are required to carry the shear. To isolate them from the homogeneous simple shearing associated with the slip mode, two approaches are used in the DFT calculations of the GSFE surfaces for all five planes. The more commonly used, standard relaxation (SR), approach involves shifting the upper half of the crystal with respect to the lower half of the crystal along the glide direction in a small displacement step, and, at each displacement, minimizing the energy of the system by fixing all atomic positions in both the upper and lower crystals in the x and y directions and allowing positions in the z direction to relax^31, 32, 37^. This method prevents any additional atomic motions from occurring, apart from those required to carry the theoretical simple shear associated with the slip mode. In the second method, we call the additional relaxation (AR) approach, we allow for an additional relaxation of all atomic positions along the direction lying normal to the glide direction^36, 38, 39^.

**Figure 2:**
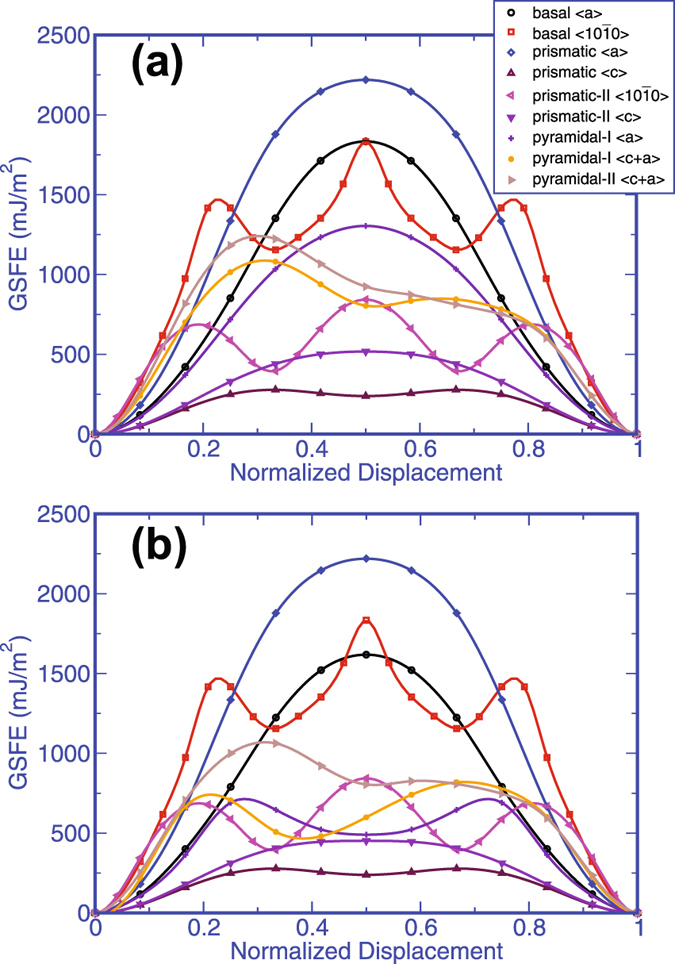
Comparison of the DFT calculated GSFE curves for all nine slip modes in *ω*-Zr obtained from the (**a**) SR calculations and (**b**) AR calculations. The x-axis displacement shift corresponds to the actual displacement along the glide direction normalized by the full Burgers vectors for each slip system.

To find the minimum energy path taken, the 2D GSFE surfaces were calculated (see supplement Figs S2 and S3). For reasons discussed below, for four out of the five planes considered, the energetically favorable pathway lies along the slip direction and hence in these cases, it suffices to evaluate the variation in the GSFE with displacement shifts along the slip direction. Figure 2 compares the calculated GSFE curves using the SR and AR approaches for the nine possible slip modes in *ω*-Zr. The abscissa is the normalized displacement, the actual displacement along the glide direction (normalized by the Burgers vector b). The periodic displacement shift across a given glide plane along the glide direction would correspond to the Burgers vector of a full dislocation for that slip mode. Often the lowest energy configuration of a dislocation core does not correspond to that of the theoretical (full) dislocation, but, for instance, to one that is dissociated into two or more partial dislocations^40^. In cases where the dissociated dislocation core configuration lies solely within the glide plane, it is possible to additionally forecast from the GSFE surfaces, the set of partial dislocations that together can produce the same total displacement shift as a full dislocation. These partial Burgers vectors would correspond to displacement shifts producing a local minimum or minima in the GSFE surface. Comparison of the SR versus AR GSFE calculations reveals that allowing for an additional relaxation in AR approach affects some, but not all, of the GSFE surfaces, suggesting that the characteristics of the local atomic accommodation mechanisms vary with slip mode. As such mechanisms enabling shearing would affect the preference for slip via that slip mode, we investigated further the atomic displacements involved in every stage of the shearing process.

We begin with basic floor planes of the hex unit cell, or formally, the basal planes. Two independent slip modes reside on the basal plane. Figure 2 shows the GSFE curves for the basal slip modes using SR and AR approaches. Compared to the other slip modes, the calculations indicate that the basal slip modes are the relatively hard systems for shearing in *ω*-Zr. Its SR GSFE curve does not exhibit local minima but that for the basal $\langle 10\bar{1}0\rangle$) slip mode possesses local minima at two displacement shifts along vectors $\frac{1}{3}\mathrm{[10}\bar{1}\mathrm{0]}$ and $\frac{2}{3}\mathrm{[10}\bar{1}\mathrm{0]}$. The AR GSFE calculation shows that relaxation of the atoms along the direction normal to glide direction slightly lowers the energy involved in the shearing process. We find that energy reduction is enabled by relatively short, uncoordinated atomic shuffles that did not occur in the simulation via the SR approach. Moreover, these shuffles accompany glide for all displacement shifts in the glide direction and are a consequence of the low symmetry atomic structure along the normal direction in the slip plane. Consequently, the shape of the energetic landscape with and without the additional relaxation are similar. Specifically, for basal $\langle 10\bar{1}0\rangle$) slip, the local minima are realized at the same displacement shifts and for basal 〈*a*〉 shuffling does not enable shearing to reach a more energetically favorable shearing pathway.

Next we consider the wall planes of the hex unit cell, which are the prismatic planes. For the hex crystal structure of *ω*-Zr, two independent slip modes reside on the prismatic plane: prismatic 〈*a*〉 and prismatic 〈*c*〉. The calculations indicate that the former mode is one of the hardest modes in *ω*-Zr, in stark contrast to its predominance in *α*-Zr^23^. As discussed in the supplement (Figs S2 and S4), we found that these are two separate glide planes for the prismatic slip system and dislocation glide along 〈*a*〉 and 〈*c*〉 would involve two different planes. Unlike the basal plane modes, shearing in the prismatic 〈*a*〉 and prismatic 〈*c*〉 does not involve atomic shuffling. This is evident since every displacement shift in the AR approach produces the same GSFE curve as the standard approach for both prismatic slip modes. This fully coordinated glide arises since the atomic positions in the prismatic plane about these glide directions are symmetric.

The hex unit cell also contains a prismatic-II plane that could support two additional slip modes, each with a crystallographically distinct slip direction (see Fig. 1a) prismatic-II $\langle 10\bar{1}0\rangle$ and prismatic-II 〈*c*〉. We find from the calculated GSFE curves that these two glide directions incur significantly different energies. Shearing via the prismatic-II 〈*c*〉 mode does not attain a local minimum but shearing along $\langle 10\bar{1}0\rangle$ encounters two local minima at displacement shifts $\frac{1}{3}\mathrm{[10}\bar{1}\mathrm{0]}$ and $\frac{2}{3}\mathrm{[10}\bar{1}\mathrm{0]}$. The reduction in energy seen under AR conditions and further analysis of the in-plane atomic displacements reveal that prismatic-II 〈*c*〉 shearing for all shifts in the glide direction is accompanied by uncoordinated atomic shuffles. As in the foregoing cases where atomic shuffling occurs, we find that this glide direction is a low symmetry glide direction of the prismatic-II plane (Fig. 2). The in-plane atomic configuration about the $\langle 10\bar{1}0\rangle$ glide direction is symmetric, and as a result, the GSFE curve from the SR and AR conditions are the same.

Next, we examine the GSFE curves associated with shearing the pyramidal-II plane along $\langle c+a\rangle =\frac{1}{3}\mathrm{[11}\bar{2}\mathrm{3]}$. As shown in Fig. 2, we find that the SR GSFE curve for this slip mode does not contain local minima; however, the AR-GSFE curve displays a local minimum at a displacement shift of $\frac{1}{6}\mathrm{[11}\bar{2}\mathrm{3]}$. In this case, additional short atomic shuffles accompanying the shearing direction result in a lower minimum energy state. The local minimum suggests that the dislocation could lower its energy by dissociating into two equal length $\frac{1}{2}\langle c+a\rangle$ partials with a stacking fault in-between with a formation energy of 803.5 *mJ*/*m* ^2^. This type of configuration could be seen in microscopy if high-pressure probing were possible; however, direct experimental observations of individual dislocations in deformed *ω*-Zr have not yet been reported. It should be mentioned that AR GSFE curves for this same slip mode in hcp Mg exhibit the same local minimum, and in corroboration, a equally split core configuration for pyramidal-II 〈*c* + *a*〉 dislocations has been seen both experimentally and in atomic-scale simulation^41–44^.

**Figure 3:**
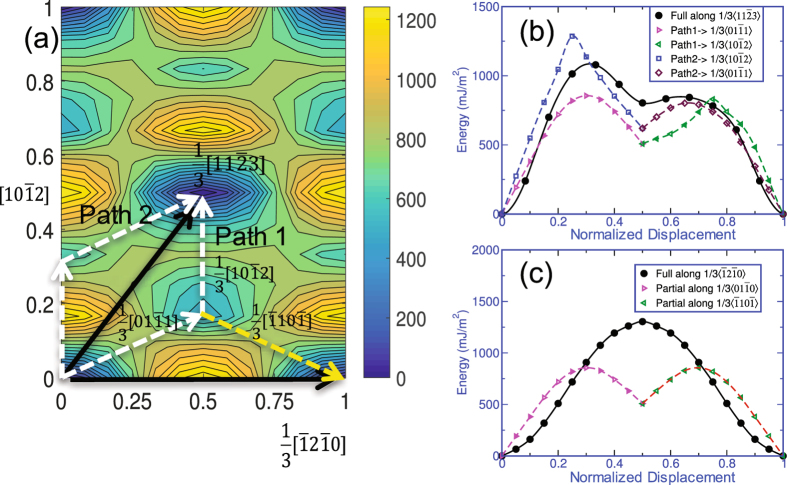
(**a**) Two-dimensional *γ*-surface for the pyramidal-I plane in *ω*-Zr obtained from DFT calculations. (**b**) The possible partial dislocations after dissociation of a full 〈*c* + *a*〉 dislocation on the pyramidal-I plane, and (**c**) the partial dislocations after dissociation of a full 〈*a*〉 dislocation on the pyramidal-I plane. The x-axis displacement shift corresponds to the actual displacement along the glide direction normalized by the full Burgers vectors for each slip system.

Last, we consider the GSFE associated with shearing the pyramidal-I planes. This plane is atomically rumpled unlike the other planes discussed so far. For these two slip systems on the pyramidal-I plane, we find a very large difference between the SR and AR GSFE curves. For the pyramidal-I 〈*a*〉 slip mode, the SR GSFE curve does not possess local minima but the AR GSFE curve shows a local minimum at $\frac{1}{2}\langle a\rangle$. For the pyramidal-I 〈*c* + *a*〉 slip mode, the SR GSFE curve has a local minimum associated with displacement shift vector $\frac{1}{6}\mathrm{[11}\bar{2}\mathrm{3]}$ but in the more accurate AR GSFE curve, this local minimum not only experiences a substantial reduction in energy but also occurs at another shift vector. Such tremendous differences cannot be expected to result solely from the local relaxations in the atomic positions near the glide plane as in the other modes. Further analysis shows that achieving the structures resulting from the crystallographic shifts not only involves local atomic shuffling but also uniform glide of the top crystal with respect to the lower crystal along an in-plane direction normal to the shearing direction. The latter glide motion is relatively large, indicating that the shear path of minimum energy must lie in-between these two in-plane glide directions. To find the local minimum, we calculated the two-dimensional (2D) GSFE surface, which is shown in Fig. 3(a). This surface reveals that the local minimum on the pyramidal-I plane is achieved at a displacement shift of $\frac{1}{6}\mathrm{[01}\bar{1}\mathrm{1]}$, as indicated by the dotted vector in Fig. 3. This shift implies that a full 〈*c* + *a*〉 dislocation can lower its energy by dissociating into two partial dislocations on the pyramidal-I plane with Burgers vectors $\frac{1}{3}\mathrm{[11}\bar{2}\mathrm{3]}=\frac{1}{3}\mathrm{[01}\bar{1}\mathrm{1]}+\frac{1}{3}\mathrm{[10}\bar{1}\mathrm{2]}$. The stacking fault that lies between them would have a formation energy of 479 *mJ*/*m* ^2^. Likewise, a full 〈*a*〉 dislocation could achieve a lower energy configuration by dissociating into two equal length partials of Burgers vectors $\frac{1}{3}[\bar{1}2\bar{1}\mathrm{0]}=\frac{1}{3}\mathrm{[01}\bar{1}\mathrm{1]}+\frac{1}{3}[\bar{1}10\bar{1}]$ with the same formation energy of 479 *mJ*/*m* ^2^.

Towards determining favorability, we first rank these modes based on the peak energy values from the AR GSFE curves from the lowest (most favorable) to the highest in Table 3. From these GSFE curves, we also estimate the ideal shear stress via $\frac{1}{b}\frac{\partial \gamma }{\partial u}$ taken from the lower of the two peaks obtained from either shifting from left to right or in reverse from right to left on the GSFE curves^45^ and list this on Table 3. Based on a comparison of the ideal shear stress, the easier slip modes for accommodating 〈*c*〉 deformation would be prismatic 〈*c*〉 and pyramidal-II 〈*c* + *a*〉 and those for accommodating 〈*a*〉 deformation would be prismatic-II $\langle 10\bar{1}0\rangle$.

**Table 3:** Values of the low-energy peak (in *mJ*/*m* ^2^) from the AR GSFE curves and the ideal shear stress (ISS) in GPa obtained from the first peak of $[\frac{1}{b}\frac{\partial \gamma }{\partial u}]$ for all slip systems at P = 0 and P = 4.9 GPa.

	P = 0 GPa		P = 4.9 GPa	
	*γ*_*peak*_	ISS	*γ*_*peak*_	ISS
prismatic 〈*c*〉	278	3.84	318	4.22
prismatic-II 〈*c*〉	444	4.95	518	5.60
prismatic-II $\langle 10\bar{1}0\rangle$	682	6.05	754	6.73
pyramidal-I 〈*a*〉	713	7.00	758	7.38
pyramidal-I 〈*c* + *a*〉	815	6.48	875	7.07
pyramidal-II 〈*c* + *a*〉	826	6.26	913	6.80
basal $\langle 10\bar{1}0\rangle$	1456	10.99	1604	11.87
basal 〈*a*〉	1607	9.85	1786	10.90
prismatic 〈*a*〉	2213	14.89	2433	16.93

**Figure 4:**
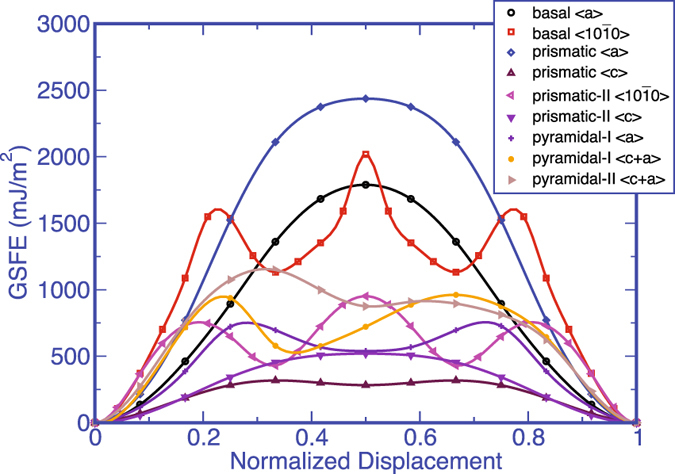
GSFE curves for all nine slip systems in *ω*-Zr obtained from the AR calculations at P = 4.9 GPa. The x-axis displacement shift corresponds to the actual displacement along the glide direction normalized by the full Burgers vectors for each slip system.

The GSFE curves for the *ω*-Zr shown in Fig. 2 are calculated at zero pressure. To understand the effect of pressure on slip mode selection, we calculated the GSFE curves using lattice parameters corresponding to 4.9 GPa hydrostatic pressure (a pressure value above the *α* to *ω* phase transition pressure at low temperature^2, 46^). A direct comparison of GSFE curves at P = 0 and P = 4.9 GPa would provide the effect of pressure on the relative energetics of these slip systems in *ω* phase. Earlier studies have shown that the energy barrier for the *ω* to *α* phase transformation is relatively large in Zr^47^ and therefore the system would not undergo a phase transformation for these two pressures in our DFT calculations and would remain in the *ω* phase. To calculate the GSFE at 4.9 GPa, first we calculate the structural parameters (*a* and *c*) in bulk by applying the hydrostatic pressure of 4.9 GPa. Then, we use these calculated *a* and *c* parameters to construct the supercell corresponding to this elevated pressure value. During the calculation of the GSFE curve, the in-plane lattice parameters of the supercell are fixed for both the AR and SR methods. As we need to have a vacuum layer in the periodic supercell in DFT, we also we fix the z coordinates of the two outer layers in the upper and lower crystals during the elevated pressure GSFE calculations. The results are also provided in Table 3. It is found that while pressure increases the value of the fault energy, the nature of the GSFE curves does not change (see Fig. 4), suggesting that the preferred slip mode in *ω* − Zr would not change under the pressures.

## Discussion

Our predictions are consistent with the very few experimental studies that have been carried out to identify the major slip modes in *ω*-Zr. In an earlier work, Wenk *et al*.^2^ reported that using the prismatic-II $\langle 10\bar{1}0\rangle$ and basal $\langle 10\bar{1}0\rangle$ slip modes within a visco-plastic self-consistent (VPSC) model could match the measured deformation texture. Recently, N. Adachi *et al*.^48^ conjectured that prismatic-II 〈*c*〉 would achieve consistency with their experimental deformation texture. While deformation textures are sensitive to slip mode activity, the same deformation texture could easily be achieved by different amounts of strain, deformation histories, and combinations and relative amounts of slip modes. More importantly, each of the foregoing sets can only accommodate deformation in one lattice direction, either 〈*a*〉 or 〈*c*〉, and thus theoretically could not carry plasticity for a general state of strain or stress on the crystal.

**Figure 5:**
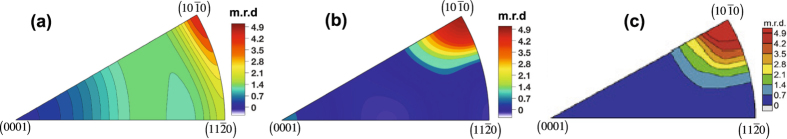
Inverse pole figure of the deformation texture of *ω*-Zr at 50% compression from the mean-field polycrystal modeling (**a**,**b**); and from the experiment (**c**) reproduced from Wenk *et al*.^2^. Critical resolved shear stress (CRSS) ratios are (**a**) all 1.0 and (**b**) are 1.0, 2.6, 2.8, and 5.0 for the prismatic-II $\langle 10\bar{1}0\rangle$, prismatic 〈*c*〉, pyramidal-I 〈*a*〉, and pyramidal-II 〈*c* + *a*〉 modes respectively.

The DFT calculations reported here have established the available slip families and imply that their activation barriers would not be the same. To demonstrate their utility, we carried out effective-medium VPSC calculations to predict the evolution of texture within an *ω*-Zr polycrystal under compression and compare it with experimental measurements from Wenk *et al*.^2^, replicated in Fig. 5(c). In this type of crystal plasticity model, the available set of slip modes would need to be assigned *a priori*, information for which the present DFT calculations can provide. Based on the ISS results in Table 3, we make available for the VPSC calculation the four slip families with the lowest ISS. Notably together these chosen four slip modes would accommodate a general deformation state (that is, those that together accommodate *a* and *c* axis). The VPSC calculation also needs as input, values for the critical resolved shear stress (CRSS) among these modes, information that is not expected of DFT. The CRSS represents an effective resistance to dislocation glide. In the VPSC calculation, the starting microstructure consists of 5000 *ω*-Zr spherical grains with a uniform distribution of lattice orientations, i.e., a so-called random texture. The calculation evolved the texture from this initial state under a uniformly applied compressive strain up to up to 50% strain in 0.1% strain increments. In these calculations, the CRSS ratios remain constant throughout the deformation.

We first make available these slip systems with equal values of CRSS, which amounts to inputting equal ratios 1:1. The results are reported in Fig. 5(a). Clearly the agreement between the calculated and experimental texture is poor. Based on this result, we then fit the ratios of the CRSS to match the texture data, using procedures common for this type of model^26^. They are usually applied to materials that deform by multiple slip modes and involve changing the ratios among the chosen set of multiple slip families until the texture matches^49–54^. Figure 5(b) shows the calculated texture using CRSS ratios of 1.0, 2.6, 2.8, and 5.0 among the prismatic-II $\langle 10\bar{1}0\rangle$, prismatic 〈*c*〉, pyramidal-I 〈*a*〉, and pyramidal-II 〈*c* + *a*〉 slip modes respectively. The result is notably consistent with the experimental texture (Fig. 5(c)). Although the fitted CRSS values do not follow exactly the same order as the DFT-derived ISS values in Table 3, they both suggest that the two easiest 〈*c*〉 and 〈*a*〉 slip families are prismatic 〈*c*〉 and prismatic-II $\langle 10\bar{1}0\rangle$, respectively, and the next easiest slip families are pyramidal-I 〈*a*〉 and pyramidal-II 〈*c* + *a*〉. As the analysis here has indicated, *ω*-Zr deforms by multiple slip families, bearing different threshold stresses to activate them. As a result, an expanded crystal plasticity modeling effort is recommended for characterizing the threshold values involving simultaneously fitting multiple stress-strain curves corresponding to distinctly different loading states.

In summary, using first-principles density functional theory calculations, we identified the preferred slip modes in *ω*-Zr by calculating the GSFE curves for nine geometrically possible slip modes. The modes with the lowest energy barriers are prismatic 〈*c*〉, prismatic-II $\langle 10\bar{1}0\rangle$ and pyramidal-II 〈*c* + *a*〉. Together, these three modes can accommodate any arbitrary crystal plastic deformation. The analysis also suggests that the low-energy configurations for dislocations on the pyramidal-I and II planes could be extended, consisting of two equal-length partials separated by a stacking fault. Using an effective-medium crystal plasticity model, we have attempted to verify whether the DFT based identified slip modes can accommodate the plastic deformation of *ω*-Zr. Both DFT and an initial effort using VPSC suggest that the activation barriers among these modes would differ, implying that *ω*-Zr will intrinsically exhibit plastic anisotropy in its plastic deformation behavior. Along these lines we have demonstrated the preferences of prismatic 〈*c*〉 and prismatic-II $\langle 10\bar{1}0\rangle$ modes with a secondary preference for the pyramidal-I 〈*a*〉, and pyramidal-II 〈*c* + *a*〉 modes. Our calculations also suggest that the predominance of these modes is not affected by pressures up to 4.9 GPa. Results on the preferred crystallographic slip modes are fundamental for understanding the plastic deformation behavior of *ω*-Zr and a composite of *α*-Zr containing retained *ω* phase.

## Methods

### Density Functional Theory

In our DFT calculations, we use the generalized gradient approximation (GGA) for the exchange correlation functional with the Perdew-Becke-Erzenhof (PBE) parametrization^55^ as implemented in the VASP code^56, 57^. The interaction between the valence electrons and ionic cores is treated using PAW potential^58, 59^. The number of valence electrons in the PAW potential for Zr is four (5s^2^, 4d^2^). We used a plane wave energy cutoff of 350 eV, and optimized the atomic structure until the force on each atom is smaller than 0.01 eV/*Å*. We used 19 × 19 × 11 and 19 × 19 × 25 Γ-centered Monkhorst Pack^60^ k-point mesh to integrate the Brillouin Zone of the hcp primitive unit cells to calculate the lattice and elastic constants of *α*-Zr and *ω*-Zr respectively. For the calculation of the elastic constants, we used finite difference distortions of the lattice as implemented in the VASP (version 5.3, ISIF = 6) with a distortion step of 1.5% strain to calculate the elastic constants for the systems under zero pressure as well as under hydrostatic pressure. 4s and 4p semi-core electrons are not included in the valence states because the pressures associated with the phase transformation are not very high, 2–4 GPa^2, 4^. To confirm, however, we repeated the lattice parameter and elastic constant DFT calculations, including 12 valence electrons, over a wide range of pressures, from −8 to 20 GPa, and found minute changes for the small pressure range (see results in the supplement Fig. S5). As a further check, additional 12 valence electron calculations were carried out for the GSFE curves for the prismatic 〈*c*〉 and prismatic-II 〈*c*〉 slip modes and the results show negligible differences with the 4-valence electron calculations (see supplement Fig. S6).

### VPSC Modeling

The effective medium VPSC model was used to simulate the evolution of texture within a polycrystal under compression to 50% strain. The starting polycrystal model consists of 5000 *ω*-Zr grains with a uniform distribution of crystal orientations, i.e., a uniform random texture. Possible changes in CRSS ratio with strain are not taken into account so these CRSS ratios remain constant throughout deformation. The imposed plastic deformation is accommodated through prismatic-II $\langle 10\bar{1}0\rangle$, prismatic 〈*c*〉, pyramidal-I 〈*a*〉, and pyramidal-II 〈*c* + *a*〉 slip modes. The CRSS ratios provided to the calculation in (b) are not from DFT but characterized to achieve consistency with the experimentally measured texture.

## Electronic supplementary material

Supplementary Materials
