# Supplementary material for: First-principles study of crystallographic slip modes in ω-Zr
Source: Sci Rep. 2017 Aug 21;7:8932. doi: 10.1038/s41598-017-09153-w (PMC5566412; doi:10.1038/s41598-017-09153-w)
Supplement: Supplementary file 1 — Supplementary Materials [file 41598_2017_9153_MOESM1_ESM.pdf]

Supplementary Materials

First-principles study of crystallographic slip modes in ω-Zr

Anil Kumar[1], M. Arul Kumar[2], and Irene J. Beyerlein[3]

[1]Theoretical Division, Los Alamos National Laboratory, Los Alamos, NM 87545
[2]Materials Science and Technology Division, Los Alamos National Laboratory, Los Alamos, NM 87545
[3]Mechanical Engineering Department, Materials Department, University of California at Santa Barbara, Santa Barbara, CA 93106

1. The Supercell models for different slip systems

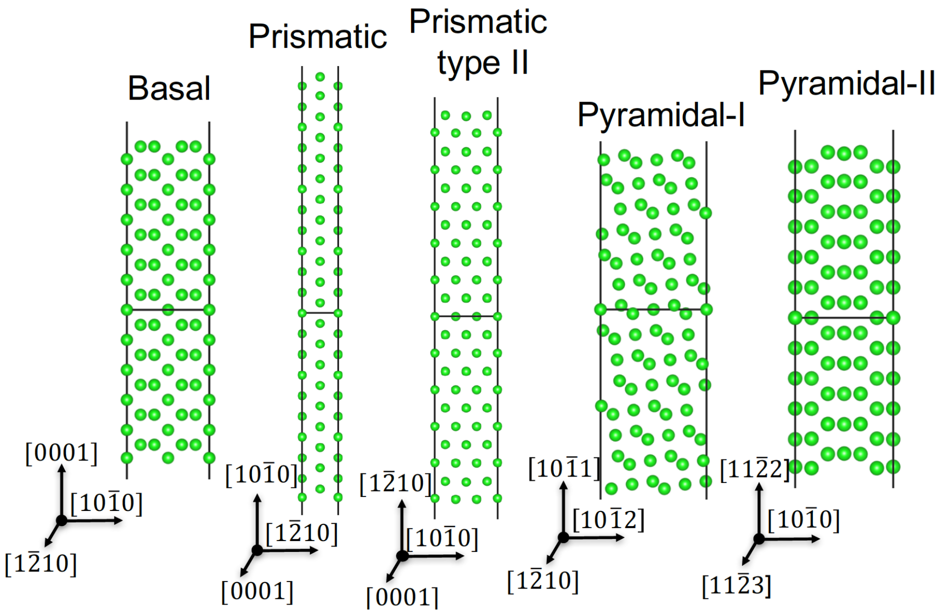

Figure S1: The supercells used for calculating the GSFE for the five slip planes. Their crystallographic orientations are provided. The supercells are periodic along all three directions. For each model, the direction normal to the slip plane contains a ~15Å vacuum layer.

2. The 2D GSFE surfaces

In this work, we calculated the 2D GSFE surfaces for all five planes. Of interest here are local minima on these surfaces and the in-plane shear displacements needed to reach these minima, These two factors are governed by the symmetry of the atoms about the glide directions. Only one of the five planes, the pyramidal-I plane, possesses an asymmetric arrangement of atoms

within the glide plane, and for this reason, we choose to include the full 2D surface for this plane in the main text. For the other four planes, the atomic arrangement is symmetrical about the theoretical shear direction and hence the low energy pathway corresponds to the theoretical shear direction. It, therefore, is sufficient to show in the main text, only the GSFE curve along this direction. However to be complete, in this supplement, in Figs. S2 and S3 below, we present the 2D surfaces we calculated using the same method for these four planes.

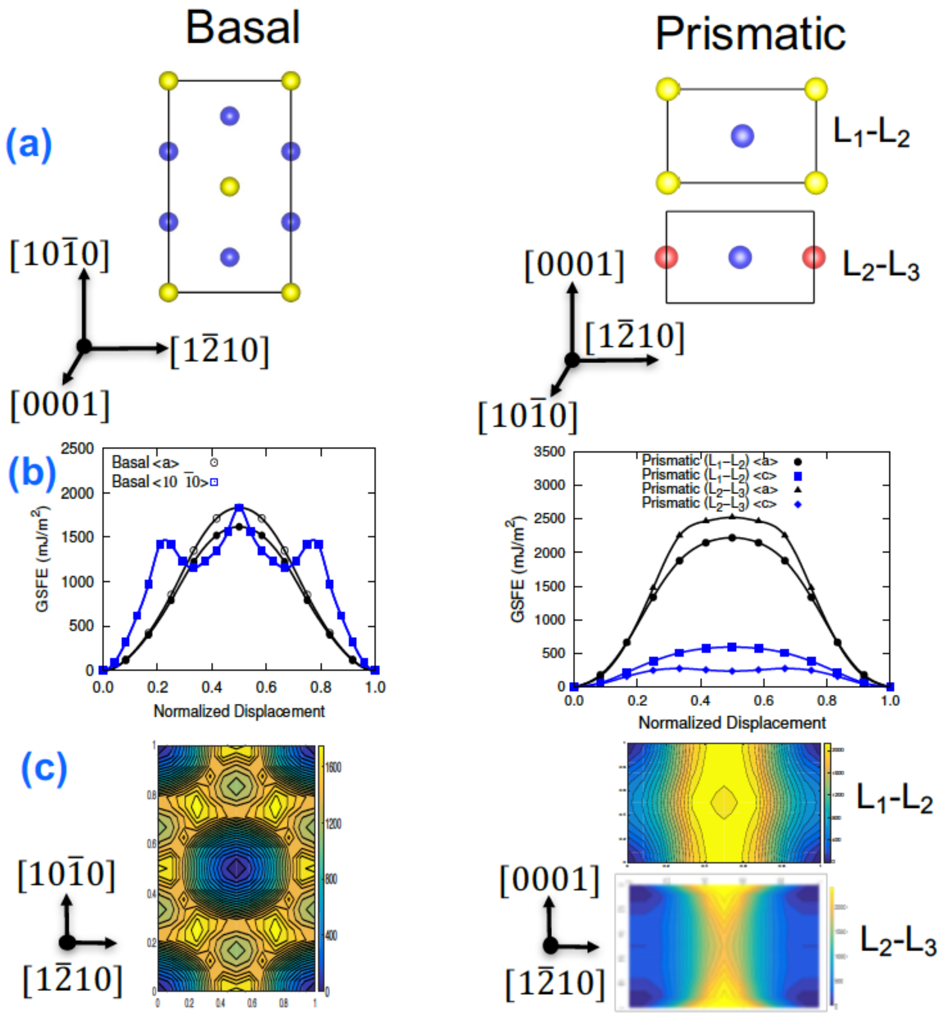

Figure S2: Calculated 2D GSFE energy surfaces for the basal and prismatic slip planes. The top row (a) shows the atomic structures for these slip planes. Here, we only show the atomic positions in the two atomic layers near the glide plane: one below the glide plane (atoms shown in yellow) and the other above the glide plane (atoms shown in blue). For the prismatic slip, there are two glide planes between layers L1-L2 and L2-L3 [atoms in Layer L1, L2, L3 are yellow, blue and red respectively]. We found that dislocation glide is energetically favorable between layers L1 and L2 along the direction a, and between layers L2 and L3 along direction c on the prismatic plane. Accordingly, we studied the GSFE surfaces for both the glide planes associated with the prismatic slip modes. In the second row (b), we show the GSFE curves calculated along selected directions for these planes using SR and AR methods. The data for the SR method are

shown by the open symbols and data for the AR method are shown by the filled symbols. For the structures, where the SR and the AR methods give the same GSFE, we only see the filled symbols. The third-row (c) shows the 2D GSFE surface calculated using DFT on a grid for the two slip planes.

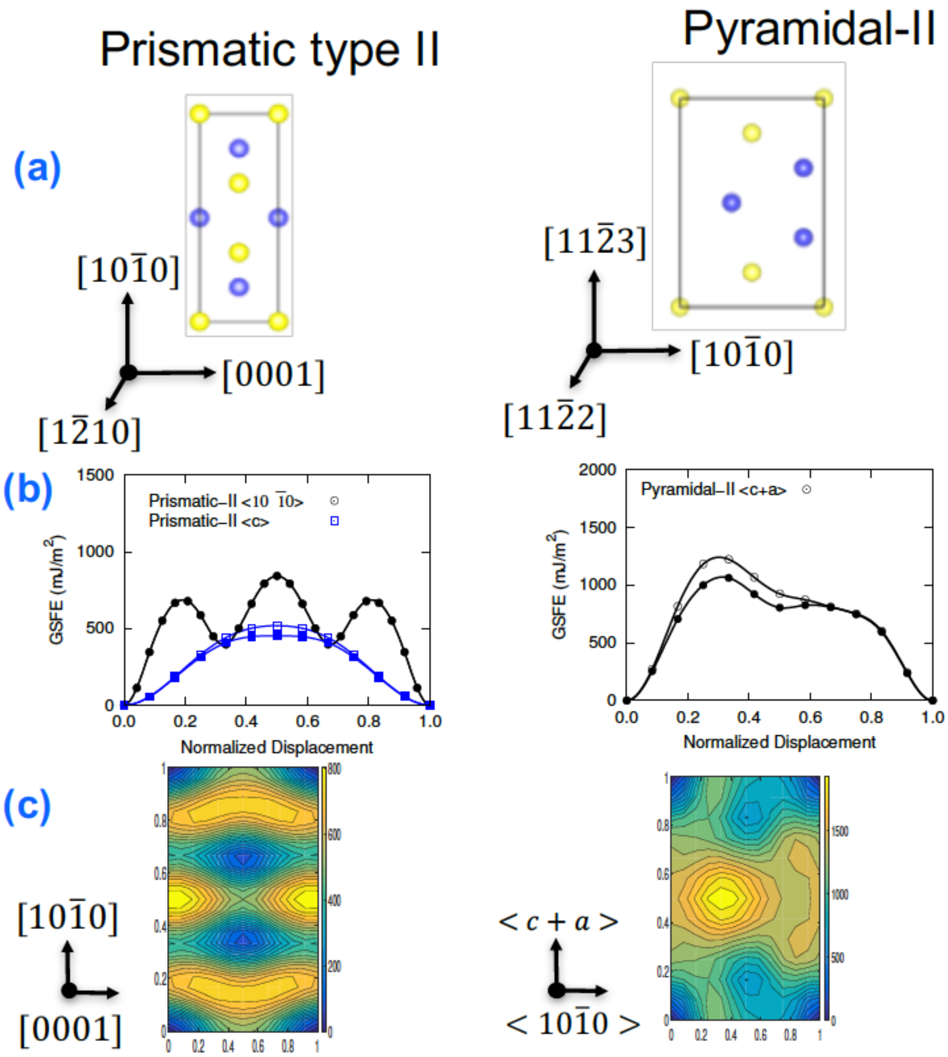

Figure S3: Calculated GSFE energy surfaces for the prismatic-II and pyramidal-II slip planes. Similar to figure S2, the top row (a) shows the atomic structures for the two slip planes. In the second row (b), we show the GSFE calculated along selected directions using SR and AR methods. The data for the SR method are shown by the open symbols and data for the AR method are shown by the filled symbols. The third-row (c) show 2D GSFE surface calculated using DFT on a grid for the two slip planes.

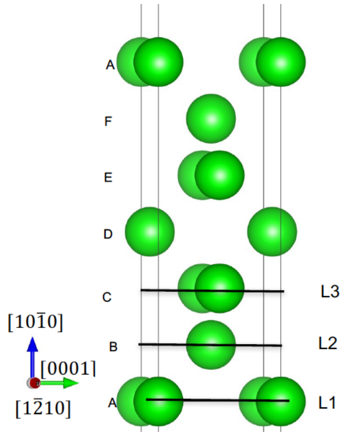

Figure S4: The stacking ABCDEF along the normal direction $[10\bar{1}1]$ *for the prismatic plane in omega Zr. The inter-planar spacing between the different layers along* $[10\bar{1}1]$ *direction is the same. However, the atomic structure for glide along the <a> and <c> directions are different for the glide between layers L1 and L2 and layers L2 and L3.*

As shown in Figure S4, there are two glide planes for the prismatic plane. Based on calculated GSFE for these two glide planes for the prismatic slip system, we found that it is easy for the <a> dislocation to glide between layers L1 and L2 and the <c> dislocation between layers L2 and L3. Interestingly, we find that dislocation glide on the prismatic plane along the <a> and <c> directions would involve two different planes. As the atomic structure is symmetric for the prismatic plane, the standard and fully relaxed methods give the same GSFE curves. The calculated 2D GSFE surfaces (see row c in Figure S2) for these two planes do not suggest any other lower minimum energy pathway for the prismatic plane.

In figure S3, first we provide the calculated GSFE along the two normal directions (row b) using the SR and AR methods and 2D GSFE surface (row c) for the prismatic-II plane. The SR and AR methods give the same energy along $[10\bar{1}1]$ as the structure normal to this line is highly symmetric. We find that relaxation normal to glide along the [0001] direction gives slightly lower energy compared to the standard method. The calculated 2D GSFE surface for the prismatic-II plane gives a local minimum at (0.5[0001], 0.33 $[10\bar{1}1]$). However, the analysis shows that dissociation of the full <c> dislocation into partials with this local minimum would be energetically unfavorable. Next in figure S3, we also show the 2D GSFE surface for the pyramidal-II plane. The slight change in local minimum of the GSFE along the $\frac{1}{3}[11\bar{2}3]$ for the AR method with respect to SR method is due to the local energy minimum being slightly off from the $\frac{1}{3}[11\bar{2}3]$ direction. The deviation can be found in the 2D surface. However, there is not an additional minimum energy pathway for dislocation to glide on the pyramidal-II plane.

As discussed in the main text, calculated GSFE curves using SR and AR methods for the pyramidal-I <a> slip show very large differences due to the presence of an energy minimum at ($\frac{1}{6}[\bar{1}2\bar{1}0]$, $\frac{1}{6}[10\bar{1}2]$) on the pyramidal-I slip plane as shown in the 2D GSFE plot in figure S2. This

minimum is important for the dissociation of full <a> and <c+a> dislocations on the pyramidal-I plane.

3. The valence electrons in pseudopotential

In the DFT calculations, the pseudopotential with 4 valance electrons was used. To study the properties of materials under high pressure, it can be important to also include electrons in the semi-core states as well, albiet at the expense of increased computational cost for DFT calculations. Since we performed calculations at either zero pressure or at very low pressure (4.9 GPa), we expect that the pseudopotential with 4 valance electrons would give nearly the same results as the pseudopotential with 12 valance electrons. To confirm, Figure S5 shows the lattice constants and elastic constants as a function of pressure for the pseudopotential with either 4 electrons or 12 electrons.

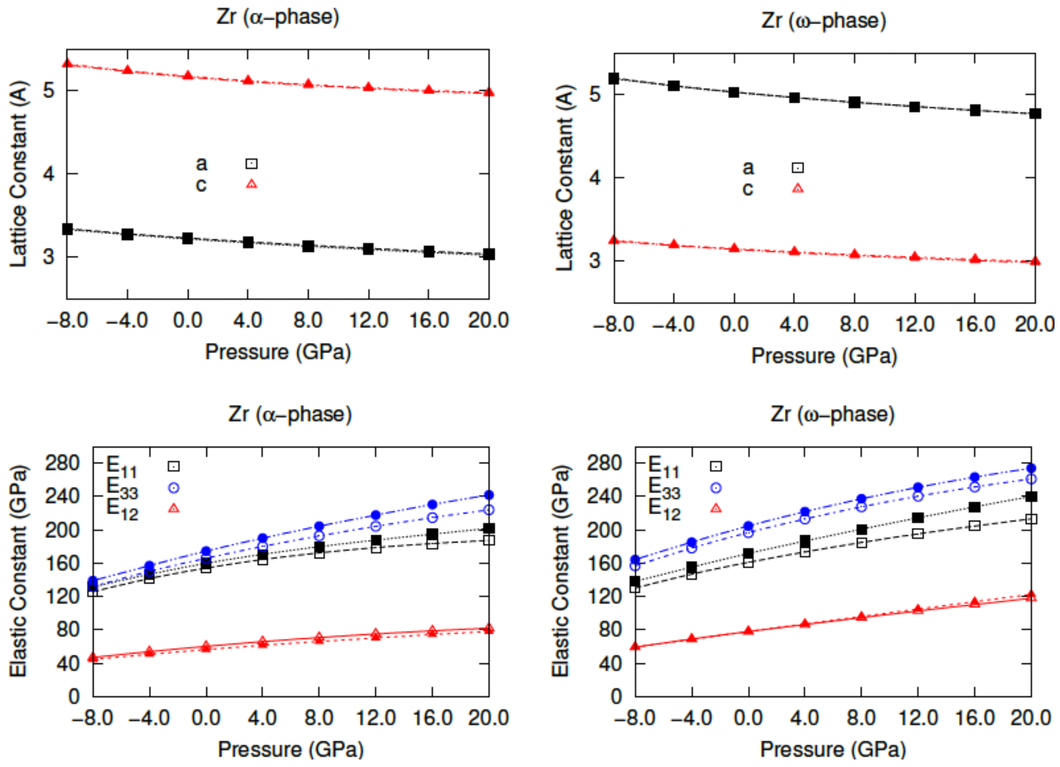

Figure S5: The calculated lattice and elastic constants for the α and ω phases of Zr using 4 and 12 valance electrons in the system. The values using 4 electrons are shown by the open symbols and the values using 12 electrons are shown by the filled symbols.

In Figure S6, we show a comparison of the GSFE curves for the two lowest energy surfaces, the prismatic <c> and prismatic-II <c> slip planes, using 4 and 12 valence electrons (see Figure S6). The results show that the relative difference between the unstable energy values on the GSFE curves does not change with number of valence electrons.

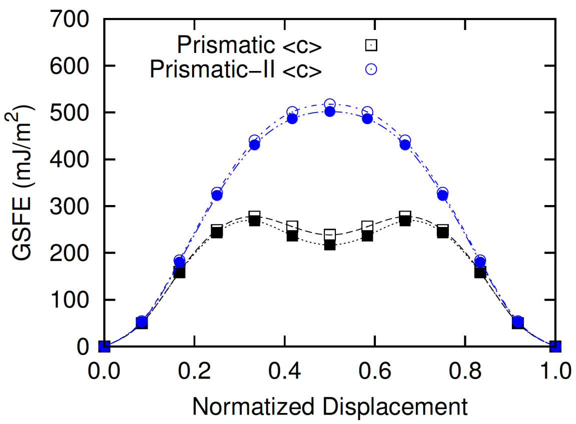

Figure S6: The calculated GSFE curves for the prismatic <c> and prismatic-II <c> slip systems in the ω phase of Zr using 4 and 12 valance electrons. The calculations using 4 electrons are shown with the open symbols and the curve using 12 electrons are shown by the filled symbols.
